# Effect of a physical activity and sleep m-health intervention on a composite activity-sleep behaviour score and mental health: a mediation analysis of two randomised controlled trials

**DOI:** 10.1186/s12966-021-01112-z

**Published:** 2021-03-25

**Authors:** Mitch J. Duncan, Anna T. Rayward, Elizabeth G. Holliday, Wendy J. Brown, Corneel Vandelanotte, Beatrice Murawski, Ronald C. Plotnikoff

**Affiliations:** 1 School of Medicine & Public Health; Faculty of Health and Medicine, Priority Research Centre for Physical Activity and Nutrition, The University of Newcastle, University Drive, Callaghan, NSW 2308 Australia; 2 Priority Research Centre for Physical Activity and Nutrition, The University of Newcastle, University Drive, Callaghan, NSW 2308 Australia; 3 School of Education, University of Newcastle, Callaghan, NSW 2308 Australia; 4 School of Human Movement and Nutrition Sciences, The University of Queensland, St Lucia, QLD 4072 Australia; 5 Physical Activity Research Group, School for Health, Medical and Applied Sciences, Central Queensland University, Rockhampton, Queensland Australia

**Keywords:** Physical activity, Sitting, Resistance training, Sleep quality, Sleep health, Depression, Anxiety, Stress, Mediation, Quality of life

## Abstract

**Background:**

To examine if a composite activity-sleep behaviour index (ASI) mediates the effects of a combined physical activity and sleep intervention on symptoms of depression, anxiety, or stress, quality of life (QOL), energy and fatigue in adults.

**Methods:**

This analysis used data pooled from two studies: Synergy and Refresh. Synergy: Physically inactive adults (18–65 years) who reported poor sleep quality were recruited for a two-arm Randomised Controlled Trial (RCT) (Physical Activity and Sleep Health (PAS; *n* = 80), or Wait-list Control (CON; *n* = 80) groups). Refresh: Physically inactive adults (40–65 years) who reported poor sleep quality were recruited for a three-arm RCT (PAS (*n* = 110), Sleep Health-Only (SO; *n* = 110) or CON (*n* = 55) groups). The SO group was omitted from this study. The PAS groups received a pedometer, and accessed a smartphone/tablet “app” using behaviour change strategies (e.g., self-monitoring, goal setting, action planning), with additional email/SMS support. The ASI score comprised self-reported moderate-to-vigorous-intensity physical activity, resistance training, sitting time, sleep duration, efficiency, quality and timing. Outcomes were assessed using DASS-21 (depression, anxiety, stress), SF-12 (QOL-physical, QOL-mental) and SF-36 (Energy & Fatigue). Assessments were conducted at baseline, 3 months (primary time-point), and 6 months. Mediation effects were examined using Structural Equation Modelling and the product of coefficients approach (AB), with significance set at 0.05.

**Results:**

At 3 months there were no direct intervention effects on mental health, QOL or energy and fatigue (all *p* > 0.05), and the intervention significantly improved the ASI (all *p* < 0.05). A more favourable ASI score was associated with improved symptoms of depression, anxiety, stress, QOL-mental and of energy and fatigue (all *p* < 0.05). The intervention effects on symptoms of depression ([AB; 95%CI] -0.31; − 0.60,-0.11), anxiety (− 0.11; − 0.27,-0.01), stress (− 0.37; − 0.65,-0.174), QOL-mental (0.53; 0.22, 1.01) and ratings of energy and fatigue (0.85; 0.33, 1.63) were mediated by ASI. At 6 months the magnitude of association was larger although the overall pattern of results remained similar.

**Conclusions:**

Improvements in the overall physical activity and sleep behaviours of adults partially mediated the intervention effects on mental health and quality of life outcomes. This highlights the potential benefit of improving the overall pattern of physical activity and sleep on these outcomes.

**Trial registration:**

Australian New Zealand Clinical Trial Registry: ACTRN12617000680369; ACTRN12617000376347.

Universal Trial number: U1111–1194-2680; U1111–1186-6588. Human Research Ethics Committee Approval: H-2016-0267; H-2016–0181.

**Supplementary Information:**

The online version contains supplementary material available at 10.1186/s12966-021-01112-z.

## Introduction

It is well established that regular participation in physical activity improves mental well-being, and reduces the risk of several chronic diseases and all-cause mortality [1, 2]. There is also robust evidence that adults who obtain 7–9 h of sleep per day have reduced risk of these same health outcomes [3, 4]. It is estimated that between 20 and 30% of Australian adults engage in a pattern of physical activity and sleep characterised by low levels of physical activity, short sleep duration and poor sleep quality [5, 6]. Physical activity and sleep can jointly influence health [6–8], though few studies to date have examined how different combinations or patterns of these behaviours influence health outcomes [4, 9, 10]. Consequently, there is a need to better understand how physical activity and sleep jointly influence health outcomes [3].

Much of the evidence to date has derived from prospective cohort studies that examined association between different combinations of physical activity and sleep duration and all-cause mortality [4, 10]. These studies used varying thresholds to classify physical inactivity (e.g., < 1 h/day, < 450 MET min/wk) and sleep duration (e.g., < 6 h/day, < 7 h/day) which limits the potential for direct comparison. However, both studies observed that participants classified as the least physically active, who also reported short sleep duration, had a significantly increased risk of all-cause and cardiovascular mortality, relative to participants classified as the most physically active who also reported mid-range sleep duration [4, 10]. Keadle and colleagues [9] extended these studies [4, 10] that examined activity–sleep dyads (i.e., duration of activity and sleep, activity duration and sleep quality) by using a behaviour index comprised of multiple indicators of physical activity (i.e., volume of light, moderate- and vigorous-intensity physical activity, duration of sitting time, frequency of resistance training) and sleep duration. Relative to participants with the lowest quintile, participants classified in the highest quintile (indicating a more favourable overall pattern of these behaviours) had a significantly lower risk of all-cause (Hazard Ratio (HR) = 0.53) and cardiovascular mortality (HR = 0.42) [9]. These observations highlight the potential benefit of interventions that aim to improve the overall pattern of activity and sleep and subsequently improve health outcomes, although to date it is unclear how changes in activity-sleep behaviours during an intervention influence health outcomes.

Physical activity and sleep health can be described using multiple dimensions of each behaviour (i.e., sedentary behaviour, activity frequency, intensity, type, duration; duration, quality, timing and restorative effects of sleep [11]). This approach may better capture the overall pattern of each behaviour. This is important as several studies report that different patterns of activity (i.e., moderate to vigorous intensity physical activity (MVPA) and resistance training [12]) or sleep (i.e., sleep duration and quality [13]) influence physical (i.e., cardiometabolic health) and mental health (i.e., depressive, anxiety) in different ways [6, 14, 15]. However, we are unaware of studies that have examined how changes in physical activity and sleep jointly influence health using multiple dimensions to represent each behaviour.

Multiple behaviour interventions that specifically target changes in both physical activity and sleep provide the opportunity to examine how changes in these behaviours are associated with health outcomes. Unlike the multitude of interventions that target changes in either physical activity [16–18] or sleep (i.e., targeting improvements in insomnia symptoms) [19, 20] there are few published interventions that have specifically targeted changes in both physical activity and sleep [21–25]. Although some interventions target other behaviours (e.g., diet [24, 25], or PAP use [23]) that make them unsuitable to pool with studies that only target activity and sleep. The Synergy and Refresh trials were separate studies that used the “Balanced” mobile health platform (m-health) to deliver a common combined physical activity and sleep intervention in two separate populations (See methods for further details). The rationale, methods and primary outcomes of these studies were described elsewhere [21, 22, 26, 27]. The current study pooled data from these studies to maximise statistical power and examine how intervention effects on mental health are mediated by changes in the overall patterns of physical activity and sleep.

**Fig. 1:**
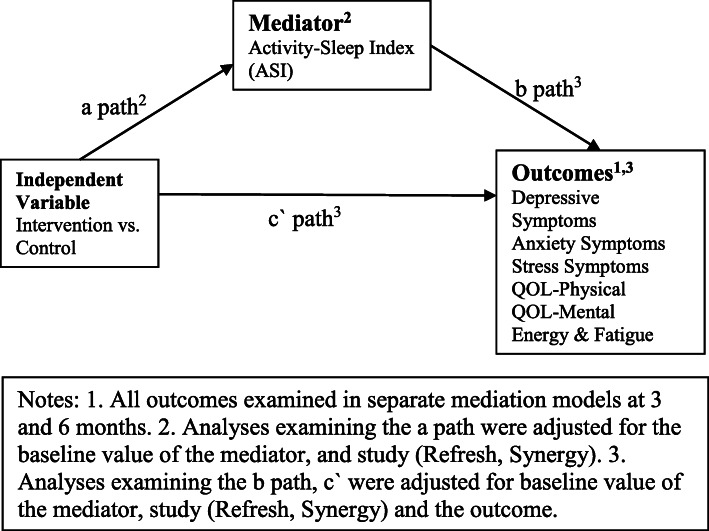
Overview of mediation model

Therefore, the aims of this study were to examine 1) the effects of a combined physical activity and sleep m-health intervention on a behavioural index (activity–sleep index) composed of multiple dimensions of physical activity and sleep health, 2) the effects of a m-health intervention on quality of life, ratings of energy and fatigue, and symptoms of depression, anxiety and stress, 3) how the behavioural index mediates the effects of a m-health intervention on these outcomes (See Fig. 1).

## Methods

### Study design

Data for this study were pooled from two separate Randomised Controlled Trials (RCT) of a m-health intervention, which was designed to improve physical activity and sleep health behaviours of physically inactive adults with poor sleep quality without a sleep disorder [21, 22]. The rationale, study protocol and design, and main outcomes of each trial are described elsewhere [21, 22, 26, 27]. Commonalties between the two studies in the physical activity and sleep health intervention, control group, outcomes assessed and assessment methods used allowed for data to be pooled for the current study as described below. Details of the inclusion and exclusion criteria specific to each study are provided in Supplementary Table 1 and summarised here. Participants were eligible if they were aged 18–55 years (Synergy Study) or 45–65 years (Refresh Study), lived in Australia, reported < 90 min of moderate to vigorous intensity physical activity in the last week, and rated their sleep quality as fairly bad or very bad. Exclusion criteria included employed in shift-work, diagnosed sleep disorder, current use of a device to track activity or sleep [21, 22]. The study design and intervention components used in each trial are shown in Supplementary Table 1.

Both studies recruited participants using primarily social media advertising. Briefly, the aim of the Synergy Study was to compare the efficacy of a combined physical activity and sleep health intervention with a wait-list control [21, 26]. Participants (*n* = 160; mean age: 41.5 (SD = 9.9); 80% female) were recruited between June–August 2017 and the study was conducted between June 2017 and February 2018. The aim of the Refresh Study was to compare the efficacy of a combined physical activity and sleep health intervention with a sleep health-only intervention and a wait-list control [22, 27]. Participants (*n* = 275; mean age: 52.0 (SD = 6.9); 83% female) were recruited between May–September 2017 and the study was conducted between June 2017 and March 2018. The combined physical activity and sleep health intervention in both trials was the same, in regards to mode of delivery, theoretical basis, educational content, and behaviour change techniques used. The sleep health-only intervention in Refresh included the same sleep health content as the combined group, but this group did not receive any intervention content or material about physical activity beyond that provided in sleep hygiene materials (e.g., being physically active can help improve sleep). Data from this arm (*n* = 110) were therefore omitted from the current study to allow direct comparison of a combined physical activity and sleep health intervention to a control group. Both studies conducted online assessments at baseline, 3 months and 6 months, were prospectively registered with the Australian and New Zealand Clinical Trials Registry and received ethical approval (H-2016-0181, H-2016-0267) at the University of Newcastle. Participants in both trials provided informed consent. Each study used computer generated permuted block randomisation to develop the randomisation sequence, with group allocation concealed in sequentially numbered envelopes. Participants were not blinded to group allocation given the nature of the interventions. At the request of the reviewers, a researcher not involved in the current study conducted a risk of bias assessment on each trial separately using the Cochrane ROB 2.0 instrument [28]. This indicates a low risk of bias overall for all domains except the measurement of the outcome (some concerns) due to participant self-reported outcomes. (see Supplementary Table 8).

### Interventions

The combined physical activity and sleep (PAS) intervention groups (*n* = 190 (Synergy: *n* = 80 + Refresh: *n* = 110)) received access to a specifically designed app (“Balanced”) developed by the investigators, which provided a platform for implementation of key behaviour-change techniques, including self-monitoring, goal setting and feedback, that have been shown to be effective [29, 30]. Detailed description of the intervention is provided elsewhere [21, 22, 26, 27] and is summarised in Supplementary Tables 1 and 1a. Participants used the app for goal setting and action planning to increase their physical activity (i.e., MVPA, step counts and resistance training) and to improve their sleep quality and sleep behaviours (stabilising bed/wake times, sleep hygiene behaviours and stress management (e.g., progressive muscle relaxation, deep breathing exercises, mindfulness). Participants were mailed a participant handbook, containing an app installation/user guide, “tools” including strategies for stress management, goal setting and action planning (followed up with a monthly electronic copy). Weekly summaries of physical activity and sleep behaviours and progress in relation to goals, based on app entries, were emailed to participants during the 3-month intervention period. Weekly educational facts were sent via text message, and text message prompts to re-engage with self-monitoring were sent if required. Wait-list control participants (*n* = 135 (Synergy; *n* = 80; Refresh; *n* = 55)) were offered full access to the intervention after the 6-month assessment.

### Measures

#### Mediators

Participants completed all online assessments using the Qualtrics_®_ survey platform at baseline, 3 months and 6 months post baseline. Sociodemographic variables (e.g., age, gender, education, chronic disease status, height and weight to calculate BMI) were assessed at baseline. The frequency (sessions/wk) and duration of moderate-to vigorous-intensity physical activity (minutes/wk) were measured using the Active Australia Questionnaire which assesses time spent walking, and in moderate- and vigorous-intensity activities during the previous 7 days [31–34]. The concurrent validity of the Active Australia Questionnaire compared to accelerometer measures of activity and test retest reliability are similar to those of other self-report instruments [31–34]. The frequency of resistance training (sessions/wk) was measured using a single item asking participants about the number of days/wk. they engaged in resistance training [21, 27]. This item has been used to assess changes in the frequency of resistance training in previous studies [21, 27]. Duration of daily sitting time (min/day) was measured using the Workforce Sitting Questionnaire, which assesses total and domain-specific time spent sitting on work-days and non-workdays [35], and has acceptable measurement properties [35]. Sleep satisfaction, daytime alertness, sleep timing, efficiency and duration were assessed using the Pittsburgh Sleep Quality Index (PSQI), which is a valid and reliable measure of sleep quality [36]. The PSQI has acceptable psychometric properties in populations, both, with and without clinical sleep disturbances [37]. The regularity of sleep was assessed using two items from the Sleep Hygiene Index, which has acceptable psychometric properties [38].

#### Activity sleep index

Consistent with the aims of the study, an activity–sleep index (ASI) was created to summarise the multiple dimensions of physical activity and sleep health. The ASI included 12 dimensions: six physical activity dimensions based on the frequency, duration, intensity and type of physical activity, and six sleep health dimensions based on the definition of sleep health [11, 39–41]. The specific items, responses, and scoring for the ASI and provided in Supplementary Table 2. The items are briefly summarised here:

1. *Frequency*–*MVPA* (Number of sessions of MVPA/wk),
2. *Frequency*–*RT* (Number of days of resistance training/wk),
3. *Intensity* (Proportion of MVPA that was vigorous in intensity),
4. *Type* (Participation in no MVPA or resistance training, either MVPA or resistance training, or both),
5. *Time* (Duration of MVPA/wk),
6. *Sitting* (Duration of sitting time/wk).
7. *Daytime alertness* (Trouble staying awake during the day),
8. *Sleep Quality* (Overall sleep quality rating),
9. *Sleep Timing* (Midpoint of sleep between 02:00 am and 04:00 am),
10. *Sleep Regularity* (Variability in bed and wake times),
11. *Sleep Efficiency* (Sleep efficiency ([sleep duration/time in bed]× 100),
12. *Sleep Duration* (Meeting age-appropriate sleep duration guidelines).

Various approaches exist to combine multiple behaviours or metrics into a single index that include time (i.e., duration of activity, sleep duration) and non-time based metrics (i.e., frequency of activity, sleep quality, timing of sleep) with varying recall periods (i.e., weekly for activity, last month for sleep quality) [42, 43]. However, there currently is no consensus on the most appropriate method. Therefore, the current study used a simple approach that rescaled each dimension from 0 to 10 with high scores reflecting lower risk behaviour, then summed each of the dimensions to form the ASI-12 (score range 0–120) at each assessment point [44]. This approach provides scores on a fixed scale (e.g., 0–10 for each dimension, 0–120 overall) that can be easily compared between studies. The approach used to rescale the individual dimensions of the ASI was $Rescaled\ score=\left(\ \frac{\left(X-{X}_{min}\right)}{X_{Range}\ }\right)n$ where X is the observed value, X_*min*_ is the minimum observed value of the original variable, X_*Range*_ is the difference between the minimum and maximum of the observed values, and *n* is upper limit of the rescaled variable (e.g., *n* = 10). Each individual dimension score was rescaled using the observed minimum and maximum of the observed values over all assessment points.

As the overall pattern of activity and sleep may be of interest in other studies that may not assess such detailed measures of these behaviours, a second index score using only 6 dimensions was also created including dimensions 2, 5, 6, 8, 11, and 12, to create the ASI-6. These items were selected a priori based on the likelihood that other studies may include similar measures and also because of their utility to capture overall patterns of activity and sleep. The ASI-6 was used in sensitivity analyses.

### Outcomes

#### Mental health and quality of life

Symptoms of depression, anxiety and stress were assessed using the depression, anxiety and stress scale (DASS-21), which has adequate psychometric properties [45]. The DASS-21 has satisfactory levels of internal consistency for each of its individual scales of depression (*r* = 0.88), anxiety (*r* = 0.82) and stress (*r* = 0.90), with higher scores indicating more severe symptoms [46]. Health-related quality of life (HRQoL) was assessed using the 12-Item Short Form Health Survey which measures self-reported physical (QOL–Physical) and mental (QOL–Mental) health components over the previous four-week period (α = 0.89, 0.76 QOL–Physical and QOL–Mental, respectively) [47, 48]. Ratings of energy and fatigue were measured using the 3-item subscale of the Rand-36 (Version 1.0) [49]. Higher scores on the QOL–Physical, QOL–Mental and RAND-36 energy and fatigue outcomes, indicate better outcomes [49].

### Statistical analysis

The Synergy and Refresh trials were each powered based on their respective primary outcomes as reported elsewhere [21, 22, 26, 27]. No a priori power calculations were conducted for the mediation analyses presented in this manuscript. Independent t-tests (continuous data) and chi-square analyses (categorical data) were employed to test for differences in sample characteristics between survey completers and non-completers of the interventions. Survey non completers were defined as individuals who did not complete either the Active Australia Questionnaire or the PSQI at the 3 month assessment. Spearman’s Rho was used to estimate correlations between the rescaled ASI–12 dimensions. To provide information regarding how the ASI–12 changed over time in response to the intervention, descriptive statistics were presented by group at each assessment point, and for the magnitude of differences between groups at 3 months and 6 months, differences were expressed using Cohen’s d [50]. Exploratory analyses were conducted to compare change in ASI–12 from baseline to 3 months according to whether participants reported changes in severity of depression, anxiety, or stress symptoms or not. Change in symptom severity was classified as improvement (i.e., a reduction of at least one level of symptom severity score (i.e., from moderate to mild or normal)), or no change/worsening (i.e., no decrease or an increase in symptom score) [45].

Structural Equation Modelling was used to examine intervention effects on the ASI-12 at 3 months, the intervention effects on the outcomes of mental health and quality of life at 3 months, and how the ASI-12 mediates the effects of a m-health intervention on these outcomes at 3 months (Fig. 1). Figure 1 outlines the mediation model for each outcome. The a-path represents the effect of the intervention on the hypothesised mediator, the b-path represents the association between the mediator and the outcome variables and the c`-path represents the direct effect of the intervention on the outcome. Mediation effects were estimated using a product-of-coefficients approach (denoted by a*b) and the 95% confidence intervals of the *ab* coefficient were estimated using bias-corrected bootstrapping on 5000 samples. The a-path was adjusted for the baseline value of the mediator, the b-path and c-paths were adjusted for baseline value of the outcome, all analyses included an indicator to adjust for study (i.e., Synergy, Refresh). Results are reported as unstandardised coefficients. Missing data at follow up was assumed to be missing at random as age and baseline values of each outcome were associated with the likelihood of the outcome missing. Full-information maximum likelihood (FIML) was used to handle missing data and include all available observations [51]. This same analysis approach was used to examine mediation effects at 6 months, and was repeated at 3 months and 6 months using the ASI-6. Assumptions of normality, outliers and homoscedasticity for each mediation path were examined using residual plots from multiple linear regression analysis, and no noticeable violations were observed. The assumption of no exposure-mediator interaction was examined using linear regression and no significant interactions were observed. All analyses were conducted in Stata MP (15.1) using an alpha level of 0.05 to declare statistical significance.

## Results

Of the 325 participants who completed the baseline survey, 276 (84.9%) completed the 3-months survey (intervention group: *n* = 161 (84.7%); control group: *n* = 115 (85.2%)). Participant flow through each trial is shown in Supplementary Figures 1 and 2 [21, 22]. Compared with completers, non-completers of the 3-month survey were significantly younger and had significantly higher depression and stress scores at baseline (Supplementary Table 3). Participants’ baseline sociodemographic characteristics, mental health and health-related quality of life are shown in Table 1. On average, participants were aged 47 years (range 19 to 64), had a mean BMI of 28, and 81.2% were female; the majority of participants reported symptoms of depression, anxiety or stress that were classified as normal.

**Table 1:** Summary of baseline sociodemographic and mental health characteristics of participants by control and intervention groups

	Control N = 135	Intervention N = 190	Total ***N*** = 325
	M (SD), n (%)	M (SD), n (%)	M (SD), n (%)
Age (years)	46.2 (10.4)	47.2 (9.7)	46.8 (10.0)
Sex			
male	26 (19.3%)	35 (18.4%)	61 (18.8%)
female	109 (80.7%)	155 (81.6%)	264 (81.2%)
Education (years)	16.3 (2.9)	16.1 (2.8)	16.2 (2.8)
Occupation			
Blue Collar	5 (3.7%)	4 (2.1%)	9 (2.8%)
White Collar	18 (13.3%)	37 (19.5%)	55 (16.9%)
Professional	85 (63.0%)	107 (56.3%)	192 (59.1%)
Retired, Student, Home Duties, Other	27 (20.0%)	42 (22.1%)	69 (21.2%)
Income/Yr			
≤ $30,000	32 (23.7%)	30 (15.8%)	62 (19.1%)
$30,001–$50,000	14 (10.4%)	26 (13.7%)	40 (12.3%)
$50,001–$70,000	26 (19.3%)	39 (20.5%)	65 (20.0%)
$70,001–$100,000	25 (18.5%)	47 (24.7%)	72 (22.2%)
≥ $100,001	30 (22.2%)	30 (15.8%)	60 (18.5%)
don’t know/prefer not to answer	8 (5.9%)	18 (9.5%)	26 (8.0%)
BMI	27.7 (4.1)	28.5 (4.3)	28.2 (4.2)
DASS-21 Depression	11.5 (8.5)	10.1 (7.7)	10.7 (8.1)
DASS-21 Depression Category			
Normal	62 (46.3%)	99 (52.1%)	161 (49.7%)
Mild	24 (17.9%)	31 (16.3%)	55 (17.0%)
Moderate	33 (24.6%)	44 (23.2%)	77 (23.8%)
Severe	9 (6.7%)	10 (5.3%)	19 (5.9%)
Extremely severe	6 (4.5%)	6 (3.2%)	12 (3.7%)
DASS-21 Anxiety	6.6 (5.9)	5.6 (5.1)	6.0 (5.5)
DASS-21 Anxiety Category			
Normal	83 (61.5%)	134 (70.5%)	217 (66.8%)
Mild	11 (8.1%)	17 (8.9%)	28 (8.6%)
Moderate	32 (23.7%)	29 (15.3%)	61 (18.8%)
Severe	6 (4.4%)	4 (2.1%)	10 (3.1%)
Extremely severe	3 (2.2%)	6 (3.2%)	9 (2.8%)
DASS-21 Stress	14.9 (7.1)	13.7 (6.2)	14.2 (6.6)
DASS-21 Stress Category			
Normal	73 (54.1%)	117 (61.6%)	190 (58.5%)
Mild	32 (23.7%)	34 (17.9%)	66 (20.3%)
Moderate	21 (15.6%)	32 (16.8%)	53 (16.3%)
Severe	7 (5.2%)	7 (3.7%)	14 (4.3%)
Extremely severe	2 (1.5%)	0 (0.0%)	2 (0.6%)
Energy & Fatigue	45.8 (19.3)	43.9 (18.3)	44.7 (18.7)
QoL Physical Health	47.8 (6.7)	46.8 (7.4)	47.2 (7.2)
QoL Mental Health	41.4 (10.3)	42.5 (10.1)	42.0 (10.2)

Data pooled from the control and Physical Activity and sleep interventions from the pooled from Synergy and Refresh trials

Small to moderate magnitude correlations were observed between each of the ASI dimensions and also with the overall ASI-12 score (Supplementary Table 4). The ASI-12 score and ASI dimensions were similar in the intervention and control groups at baseline; higher scores were observed in the intervention group at 3 and 6 months relative to the control group (Table 2). The magnitude of between group differences in the overall ASI score at 3 and 6 months was moderate (*d* = 0.42–0.51) and ranged from small to moderate for the individual ASI dimensions (Table 2).

**Table 2:** Descriptive statistics of ASI-12 and Sub-component Scores by group and assessment point

	Baseline		3 Months			6 Months		
	Control (***n*** = 135)	Intervention (***n*** = 190)	Control (***n*** = 112)	Intervention (***n*** = 160)	Group Difference	Control (***n*** = 96)	Intervention (***n*** = 117)	Group Difference
	M (SD)	M (SD)	M (SD)	M (SD)	d (95% CI)^**a**^	M (SD)	M (SD)	d (95% CI)^**a**^
**ASI-12 Score**	46.83 (10.03)	47.83 (11.56)	51.99 (12.71)	57.89 (15.08)	0.42 (0.17, 0.66)	51.40 (12.28)	58.34 (14.60)	0.51 (0.24, 0.78)
**MVPA Frequency Score**	1.00 (1.13)	0.91 (0.80)	1.42 (1.19)	1.69 (1.48)	0.19 (−0.05, 0.43)	1.31 (1.10)	1.53 (1.31)	0.18 (− 0.10, 0.45)
**RT Frequency Score**	0.28 (0.81)	0.55 (1.48)	0.84 (1.80)	1.47 (2.05)	0.32 (0.08, 0.57)	0.63 (1.60)	1.36 (2.06)	0.39 (0.12, 0.66)
**MVPA Intensity Score**	1.65 (2.72)	1.78 (2.82)	1.99 (2.82)	2.13 (2.75)	0.05 (−0.19, 0.29)	1.99 (2.79)	2.35 (2.91)	0.13 (−0.14, 0.40)
**Activity Type Score**	5.33 (2.04)	5.53 (2.46)	5.98 (2.40)	7.06 (2.88)	0.40 (0.16, 0.64)	5.78 (2.09)	6.84 (2.91)	0.41 (0.14, 0.68)
**MVPA Duration Score**	1.25 (1.43)	0.95 (0.91)	1.90 (1.99)	2.00 (1.86)	0.05 (−0.19, 0.29)	1.99 (2.17)	2.12 (2.05)	0.06 (−0.21, 0.33)
**Sitting Duration Score**	3.42 (2.06)	3.42 (2.27)	3.62 (2.28)	3.99 (2.26)	0.16 (−0.08, 0.40)	4.33 (2.42)	4.52 (2.26)	0.08 (−0.19, 0.35)
**Sleep Alertness Score**	8.22 (2.96)	8.54 (2.48)	7.86 (3.37)	8.25 (2.82)	0.13 (−0.11, 0.37)	7.43 (3.54)	8.75 (2.50)	0.44 (0.16, 0.71)
**Sleep Quality Score**	3.51 (1.79)	3.47 (1.84)	4.46 (2.22)	5.50 (2.19)	0.47 (0.23, 0.72)	4.90 (2.21)	5.38 (2.43)	0.21 (−0.06, 0.48)
**Sleep Midpoint Score**	7.33 (4.44)	7.42 (4.39)	7.68 (4.24)	7.25 (4.48)	−0.10 (− 0.34, 0.14)	6.46 (4.81)	6.75 (4.70)	0.06 (−0.21, 0.33)
**Sleep Timing Score**	5.69 (2.32)	5.91 (2.14)	5.88 (2.20)	6.00 (1.77)	0.06 (−0.18, 0.30)	5.81 (2.08)	6.28 (1.68)	0.25 (−0.02, 0.52)
**Sleep Efficiency Score**	6.63 (2.12)	6.54 (1.80)	6.87 (2.18)	7.41 (1.92)	0.27 (0.03, 0.51)	7.03 (1.97)	7.34 (2.01)	0.16 (−0.11, 0.43)
**Sleep Duration Score**	2.52 (4.36)	2.79 (4.50)	3.48 (4.79)	5.13 (5.01)	0.33 (0.09, 0.58)	3.75 (4.87)	5.13 (5.02)	0.28 (0.01, 0.55)

All table values based on observed observations. ^a^Between groups differences at assessment point expressed as Cohen’s d are presented without adjustment for baseline value

### Mediation effects

At 3 months, there were no direct effects of the intervention on any of the mental health or quality of life outcomes (c`-path, Table 3) but there were significant total effects (c-path, Table 3) of the intervention on symptoms of stress and ratings of energy and fatigue. As shown in Table 3, the intervention significantly improved the ASI-12 at 3 months (a-path, Table 3). Higher levels (more favourable) of the ASI-12 score at 3 months were significantly associated with lower symptoms of depression, anxiety, stress, QOL mental health and ratings of energy and fatigue (b-path, Table 3). Mediation analyses revealed that the ASI-12 score significantly mediated the effects of the intervention on symptoms of depression, anxiety, stress, QOL mental health and ratings of energy and fatigue at 3 months (Table 3). The proportion of the intervention effect mediated by the ASI-12 was 55, 34, 59 49 and 43% for depression, anxiety, stress, QOL mental health and ratings of energy and fatigue respectively. At 6 months, the magnitude of associations was larger although the overall pattern of results was similar to that observed at 3 months (Table 4). Sensitivity analyses using the simpler ASI-6 revealed a similar pattern of results at both 3 and 6 months (Supplementary Tables 5 and 6).

**Table 3:** Results of mediation model examining effect of the intervention (baseline to 3 months) on ASI-12 and mental health outcomes at 3 months

Outcome	A (SE)	B (SE)	Direct (SE)	C (SE)	AB (SE)[95%CI]
Depressive Symptoms	2.99 (0.80)^a^	−0.10 (0.03)^a^	−0.25 (0.35)	− 0.56 (0.35)	−0.31 (0.12) [− 0.60,-0.11]
Anxiety Symptoms	3.01 (0.80)^a^	−0.04 (0.02)^a^	− 0.21 (0.22)	−0.32 (0.22)	− 0.11 (0.07) [− 0.27,-0.01]
Stress Symptoms	2.97 (0.80)^a^	− 0.12 (0.02)^a^	−0.26 (0.30)	− 0.63 (0.31)^a^	−0.37 (0.12) [− 0.65,-0.17]
QOL Physical Score	3.00 (0.80)^a^	0.02 (0.03)	0.28 (0.44)	0.36 (0.43)	0.07 (0.09) [−0.08,0.29]
QOL Mental Score	3.02 (0.80)^a^	0.17 (0.04)^a^	0.56 (0.57)	1.09 (0.57)	0.53 (0.20) [0.22,1.01]
Energy & Fatigue	3.01 (0.80)^a^	0.28 (0.07)^a^	1.15 (0.90)	2.00 (0.91)^a^	0.85 (0.33) [0.33,1.63]

In all models a path adjusted for study (Synergy, REFRESH) and baseline value of mediator, b path adjusted for study (Synergy, REFRESH) and baseline value of mediator and outcome. ^a^ indicates *p* < 0.05

**Table 4:** Results of mediation model examining effect of the intervention (baseline to 6 months) on ASI-12 and mental health outcomes at 6 months

Outcome	A (SE)	B (SE)	Direct (SE)	C (SE)	AB (SE)[95%CI]
Depressive Symptoms	3.07 (0.92)^a^	−0.13 (0.03)^a^	−0.31 (0.43)	−0.71 (0.44)	− 0.40 (0.18) [− 0.86,-0.13]
Anxiety Symptoms	3.12 (0.93)^a^	− 0.09 (0.02)^a^	−0.17 (0.29)	− 0.46 (0.30)	−0.29 (0.13) [− 0.70,-0.12]
Stress Symptoms	3.08 (0.92)^a^	−0.18 (0.03)^a^	− 0.40 (0.40)	−0.96 (0.43)^a^	− 0.56 (0.21) [−1.08,-0.24]
QOL Physical Score	3.00 (0.92)^a^	0.09 (0.04)^a^	0.84 (0.51)	1.11 (0.51)^a^	0.27 (0.14) [0.08,0.65]
QOL Mental Score	3.07 (0.92)^a^	0.18 (0.05)^a^	−0.11 (0.66)	0.44 (0.66)	0.55 (0.24) [0.19,1.19]
Energy & Fatigue	3.04 (0.92)^a^	0.32 (0.09)^a^	2.50 (1.17)^a^	3.48 (1.18)^a^	0.99 (0.48) [0.30,2.22]

In all models a path adjusted for study (Synergy, REFRESH) and baseline value of mediator, b path adjusted for study (Synergy, REFRESH) and baseline value of mediator and outcome. ^a^ indicates *p* < 0.05

## Discussion

This study examined the effect of a combined physical activity and sleep intervention on a physical activity-sleep index and how changes in the physical activity-sleep index mediated changes the intervention effects on mental health and quality of life. The intervention significantly improved the physical activity-sleep index and improvements in the activity-sleep index mediated intervention effects on stress symptoms and ratings of energy and fatigue. Improvements in the physical activity-sleep index also mediated improvements in symptoms of depression, anxiety and QOL mental health scores despite no significant intervention effects on each of these outcomes at 3 months. This pattern of results was similar at 6 months. These results indicate that among physically inactive adults who report poor quality sleep, the intervention improved their overall activity and sleep patterns and these improvements in contributed to improvements in indicators of mental health, energy and fatigue observed during the intervention.

Several studies have demonstrated that interventions that improve physical activity or reduce insomnia symptoms can significantly reduce symptoms of depression and anxiety [7, 16, 17, 52–55]. However, few interventions have simultaneously targeted improvements in both physical activity and sleep using dedicated intervention strategies [21, 22, 56]. The current study extends previous research by demonstrating how an intervention improves the overall pattern of activity and sleep health of physically inactive adults with poor sleep and that improvement in the overall activity-sleep pattern mediated improvements in mental health and quality of life. These findings are unique, as the activity-sleep pattern includes multiple dimensions of activity and sleep health, and improvements in the activity-sleep pattern were associated with improved mental health outcomes. The multi-dimensional nature of the index is important, given the evidence that different combinations of activity (i.e., aerobic activity and resistance training) or sleep (i.e., sleep duration and quality) [13, 57] influence health in different ways, with better health outcomes typically associated with lower risk combinations of behaviours.

Several mechanisms are thought to link physical activity (i.e., neurophysiological, psychological) and sleep disturbances (i.e., endocrine, cognitive, neuroplasticity and emotional pathways) to symptoms of depression and anxiety [58–60]; these may overlap or act in synergy to explain these findings. This study, however, could not examine the mechanisms responsible for the observed associations. The mediated effects of the intervention on mental health outcomes were larger for depressive symptoms than they were for anxiety. This is consistent with evidence of the effect of physical activity interventions on depression and anxiety [16, 17, 52], and contrary to the prospective association between insomnia symptoms and depression and anxiety [60]. These seemingly contrasting results may reflect overlapping mechanistic pathways linking physical activity and sleep with mental health and the fact that the current study examined the influence of a combined activity-sleep index rather than either behaviour separately [58–60]. Alternatively, it may reflect that the larger differences in physical activity between the intervention and control groups in the current study in comparison with the differences in sleep.

As the DASS-21 classifications are not diagnostic, the effect of improving overall activity and sleep patterns on clinical symptom severity is unknown [61]. However, the majority of participants reported normal to mild symptom severity at baseline and improvements in the activity-sleep index mediated improvements in symptom severity [61]. This is a useful finding, as even sub-clinical levels of depression and anxiety can adversely affect well-being [62, 63]. Depression, anxiety and insomnia or sleep disturbance frequently co-occur [64–66], and individuals with depression and anxiety are less active than those without these conditions [67]. Examination of comorbid depression and anxiety was beyond the scope of the current study, but would provide useful insights, as people with these chronic conditions are frequently excluded from interventions [68]. It is unclear what magnitude of improvement in the novel activity-sleep index is clinically meaningful. The exploratory analyses which compared the activity-sleep index scores of people who did or did not report improvements in their depression, anxiety and stress symptoms aimed to provide some insight into this. People who improved their depression or stress symptoms had higher ASI-12 scores than those who did not improve or remained stable (d = 0.25–0.29, Supplementary Table 7). These results require replication in a sample with a greater range of symptom severity and comparison with meaningful changes from the participant’s perspective. All outcomes examined in the current study were mental health outcomes except QOL-Physical functioning, which demonstrated both inconsistent mediation effects and intervention effects. In light of this, and the different biological mechanisms that link physical activity and sleep to physical health and mental health outcomes [3, 58, 59, 69], there is a need for more research examining the effect of the overall pattern of physical activity and sleep on physical and mental health outcomes.

Limitations of the study include the reliance on self-report measures of physical activity, sitting time and sleep, which are subject to recall bias. Objective monitoring of physical activity, sitting time and sleep using accelerometery would help to overcome. Accelerometers are useful for assessing sleep duration and timing [70], however, are unable to capture the restorative effects of sleep, which may be important for health and wellbeing. Similarly, the utility of accelerometers for assessing resistance training is limited and therefore, a combined approach is likely to be useful in future studies. The presence of undiagnosed sleep conditions (i.e., insomnia, sleep apnoea) or mental health conditions (i.e., depression, anxiety) was not assessed, so it is unclear whether these influenced the results of the current study. Multiple methods exist to combine multiple variables measured on different scales, time frames and measuring different behaviours. These methods include creating summative scores, combining standardised scores, various weighting approaches [9, 42, 43], and the use of compositional data analysis for time-based metrics [7]. The current study used a simple method to summarise the separate dimensions, but did not weight the dimensions prior to summarising them and did not examine the separate dimensions as potential mediators. The ASI is an overall score that is simple to derive, and allows multiple dimensions measured on different scales (i.e., time, frequency, or ratings) to be aggregated. Comparing different approaches to creating an overall activity-sleep index, including refining which dimensions are included, will provide greater insight into how physical activity and sleep jointly influence health. Results were based on secondary analyses of pooled data from two RCTs, consequently the current study was not specifically designed to examine how changes in activity-sleep behaviour mediated intervention effects on the outcomes examined. Additionally, the age range of participants was 18–64, although they were predominantly middle aged (Table 1), so the results are only applicable to young and mid-age adults. Participant age ranges were delimited (Synergy: 18–54 years; Refresh: 45–64) due to study design (e.g., Refresh targeting mid age adults) and pragmatic reasons (e.g., changes in sleep at older ages, different activity/guidelines for adults 65+).

In conclusion, a multiple behaviour m-health intervention that targeted physical activity and sleep with specific intervention strategies significantly improved the overall pattern of physical activity and sleep health in physically inactive adults who reported poor sleep. Furthermore, improvements in activity-sleep patterns mediated the effects of the intervention on mental health and quality of life. Interventions that create more favourable activity-sleep patterns could be useful for improving mental health and quality of life.

## Supplementary Information

**Additional file 1.**

## Data Availability

The datasets used and analysed during the current study are available from the corresponding authors on reasonable request. A public version of the Balanced app with slightly modified functionality relative to what is described in the current manuscript is available from iTunes Store (iOS) and Android Play. Content of the public version may change/ be updated over time.
