# Supplementary material for: Effect of a physical activity and sleep m-health intervention on a composite activity-sleep behaviour score and mental health: a mediation analysis of two randomised controlled trials
Source: Int J Behav Nutr Phys Act. 2021 Mar 25;18:45. doi: 10.1186/s12966-021-01112-z (PMC7992852; doi:10.1186/s12966-021-01112-z)
Supplement: Supplementary file 1 — Additional file 1. [file 12966_2021_1112_MOESM1_ESM.docx]

**Supplementary Figure 1. Study Flow for Synergy Study** (1)

**Assessed for eligibility (n = 604)**

**Excluded due to not meeting inclusion criteria (n = 420)**

Usual PA was >90 min/week (n = 128)

BMI was <18.5 (n = 11) or >35 (n = 69)

Usual sleep quality fairly/very good (n = 58)

Using sleep medication (n = 31)

Identifying as a shift worker (n = 30)

Already using an app or tracker (n = 24)

Insomnia diagnosed (n = 24)

Age was >55 years (n = 13)

Obstructive sleep apnoea (OSA) diagnosed (n = 10)

Behaviour change deemed unsafe by participant (n = 7)

Other reasons (n = 7)

Restless legs syndrome (RLS) diagnosed (n = 6)

Diagnosed for other sleep disorder/s (n = 2)

**Declined to participate (n=4)**

**Declined or discontinued intervention (n = 21)**

Sickness/injury (n = 3)

Inability to commit (n = 2)

Content not meeting expectations (n = 1)

Feeling stressed by keeping sleep logs (n = 1)

No response to survey (n = 14)

**Allocated to physical activity and sleep health intervention group (n = 80)**

**No response to 6-month survey (n = 24)**

**Declined or discontinued intervention (n = 14)**

Not satisfied with group allocation (n = 7)

No response to survey (n = 7)

**Completed 6-month assessment/analysed**

**(n = 35)**

**Eligible but did not complete baseline (n = 24)**

**Allocated to waitlist control group**

**(n = 80)**

**Completed 3-month assessment/analysed**

**(n = 59)**

**Completed 6-month assessment/analysed**

**(n = 53)**

**Completed 3-month assessment/analysed**

**(n = 66)**

**No response to 6-month survey (n = 13)**

**Completed baseline survey and randomized (n =160)**

**Supplementary Figure 2. Refresh Study participant flow (2)**


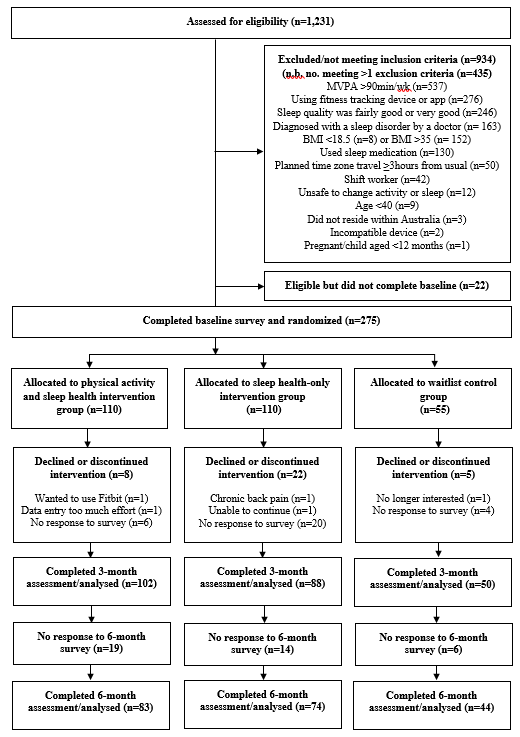


**Supplementary Table 1.** Eligibility criteria, intervention components and outcome measures used the Synergy and Refresh Studies.

| **Eligibility Criteria** | | |
| --- | --- | --- |
| Aged 18 to 55 years (Synergy Study); 40 to 65 years (Refresh Study) | | |
| Reported <90 minutes/week MVPA^a^ | | |
| Reported *fairly bad* or *very bad* sleep quality | | |
| Lived in Australia | | |
| BMI between 18.5 and 35 | | |
| Internet access and intervention app-compatible device | | |
| No previously diagnosed sleep disorder | | |
| No use of sleep medication | | |
| No pregnancy and/or no child aged ≤12 months | | |
| No conditions which would contraindicate changing physical activity level or sleep habits | | |
| Non-shift-worker | | |
| No frequent jet-lagging inducing travel | | |
| No current use of a device to track activity or sleep | | |
|  | | |
| **BCT & Intervention Components in PAS intervention *** | | |
| **Behavior Change Techniques** | **Intervention Components** | |
| Goal setting | Participants entered goals into the app for daily minutes of MVPA^a^, daily step-count, number of days/week of RT^b^, bed time and wake time as well as a number of sleep hygiene behaviors. | |
| Action planning | Participants used an action-planning “tool” provided in the Participant Handbook to plan engagement in MVPA^a^, RT^b^, and increase in step-count, use of the pedometer and sleep promoting behaviors. | |
| Self-monitoring | Participants were provided with a pedometer (Yamax SW200) to assist with self-monitoring step-count and manually logged daily minutes of MVPA^a^, number of days/week of RT^b^ , daily step-count, daily bed time and wake and the number of sleep hygiene practices implemented each week. | |
| Feedback | The app used bar graphs to show progress in relation to goals for MVPA^a^, RT^b^ , step-count, bed time/wake time, sleep duration, sleep hygiene and sleep quality over four time points; one day, one week, three-months and all. | |
|  | The app dashboard displayed immediate feedback in relation to daily minutes of MVPA^a^ and nightly sleep duration based on app entries, using a traffic light system; a green light indicated entries within 20% or meeting or exceeding goal; an orange light indicated entries between 20% and 35% of goal; a red light indicated entries more than 35% below goal. | |
|  | Participants received a weekly personalised email summarising progress in relation to personal physical activity and sleep goals based on app entries. | |
| Education | Participants accessed information relating to health benefits of physical activity and good sleep health, national physical activity and sleep duration recommendations, sleep hygiene behaviors (including stress management), goal setting, action planning and problem solving, via the app, the Participant Handbook and weekly SMS^c^ facts. | |
|  |  | |
| **Outcome measures** | | |
| **Measure** | | **Instrument** |
| Minutes of moderate- and vigorous intensity physical activity (per week)^d^ | | The Active Australia Questionnaire (AAQ) |
| Self-reported resistance training ^d^ | | Two item questionnaire asking number of days and time spent in resistance training |
| Sitting behaviour ^d^ | | The Workforce Sitting Questionnaire |
| Overall sleep health (past 30 days) ^d, e^ | | The Pittsburgh Sleep Quality Index (PSQI) |
| Health-related quality of life ^d^ | | The SF-12 |
| Energy and fatigue ^d^ | | The 3-item subscale of the Rand 36 |
| Mental health ^d^ | | The DASS-21 |
| Demographics^f^ | | Age, gender, height, weight, chronic disease status |
| Socioeconomic factors^f^ | | Education, income, marital status, occupation, working hours |
| a. MVPA = moderate-to-vigorous-intensity physical activity; b. RT = resistance training; c. SMS = short messaging service; d. PSQI sub-components include subjective sleep quality, sleep onset latency, sleep duration, sleep efficiency, sleep disturbance, sleep medication and daytime dysfunction; e. Assessed at baseline, 3 months and 6 months; f. Assessed at baseline only | | |

Note * for further details of the intervention description see supplementary table 1a.

**Supplementary Table 1a.** **Operationalisation of social cognitive factors and behaviour change strategies in the Physical Activity and Sleep Health Intervention as described in the original Synergy protocol paper. ***


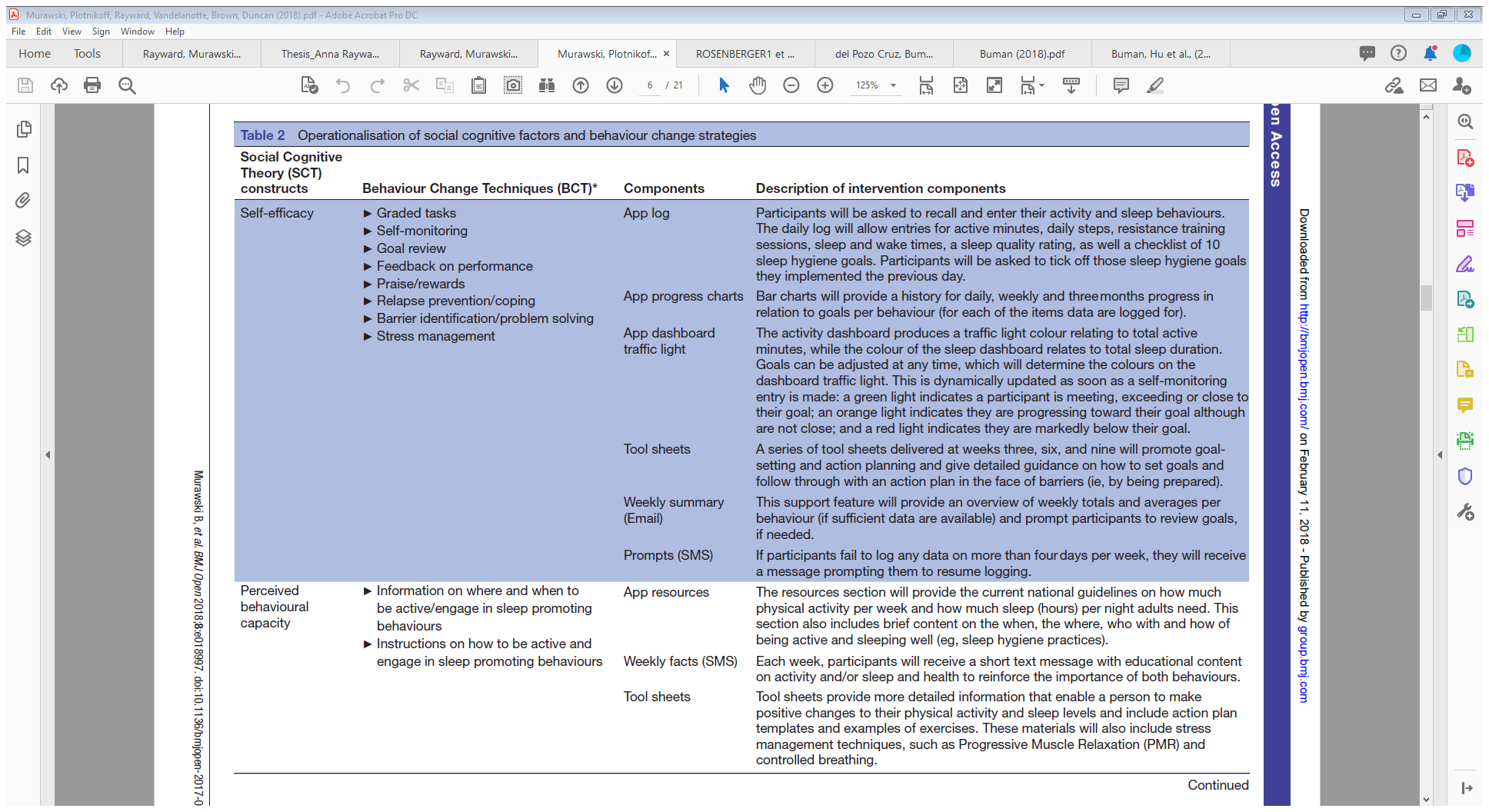


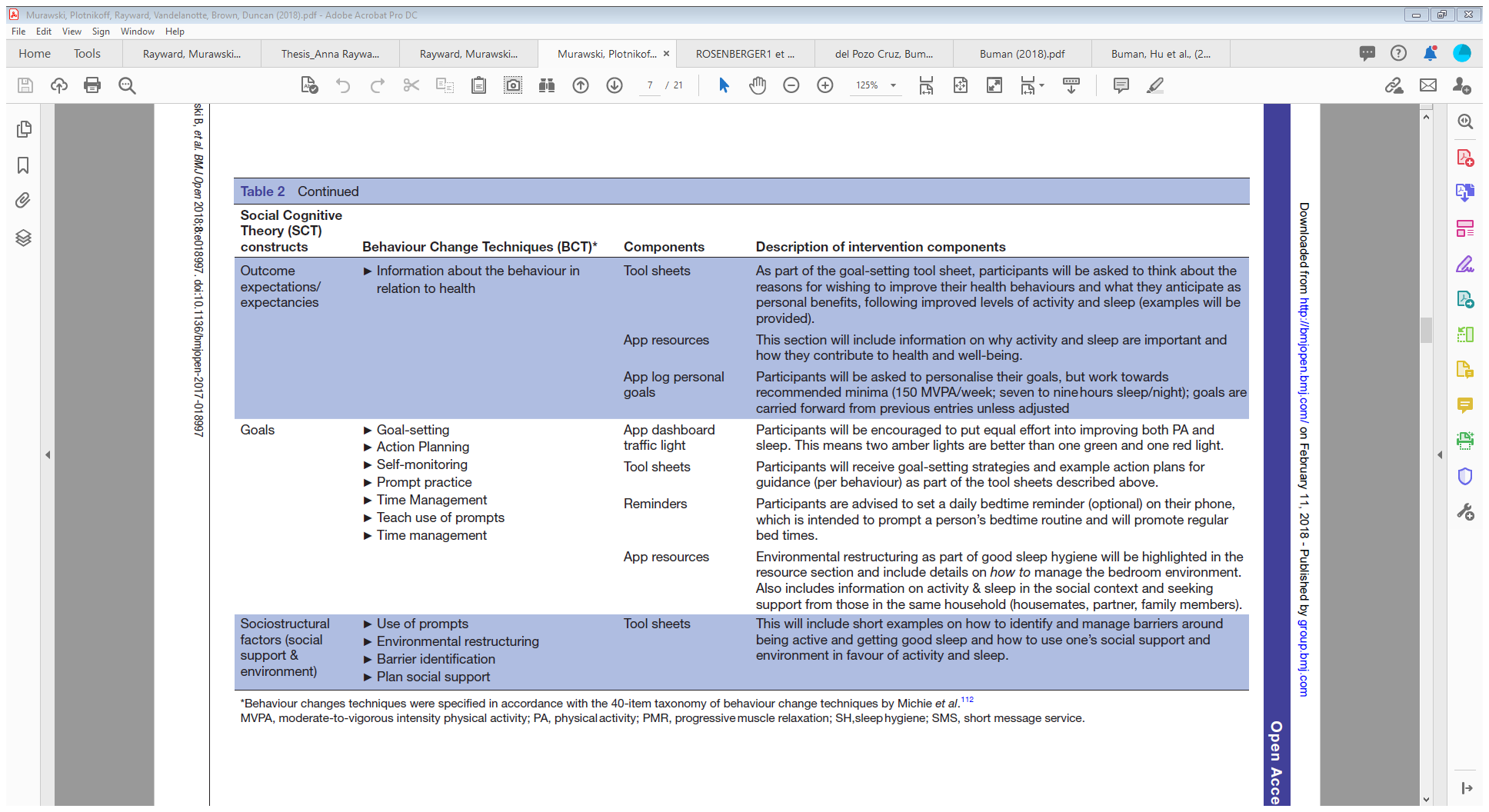


Note:* This table was originally published as Table 2 in Murawski B, Plotnikoff RC, Rayward AT, Vandelanotte C, Brown WJ, Duncan MJ (2018) Randomised controlled trial using a theory-based m-health intervention to improve physical activity and sleep health in adults: the Synergy Study protocol BMJ Open 8 doi:10.1136/bmjopen-2017-018997. Reproduced with in accordance with Creative Commons Attribution Non Commercial (CC BY-NC 4.0) license https://bmjopen.bmj.com/content/8/2/e018997.full

| **Supplementary Table 2. Description of ASI dimensions and scoring** | | |
| --- | --- | --- |
|  | **ASI-12**^1^ | **ASI-6 ^1^** |
| **Physical Activity** |  |  |
| Freq. - MVPA | No. of sessions of MVPA in last week ^2^. | - |
| Freq. – Resistance Training | No. of days of resistance training in the last week ^3.^ | No. of days of resistance training in the last week ^3.^ |
| Intensity | Proportion of vigorous intensity to total MVPA in last week ^4^. | - |
| Type | Classification based on any reported participation in MVPA and/or RT.  Scoring:  0 = No participation in MVPA or RT; 5 = Any reported participation in either MVPA or RT; 10 = Reported participation in both MVPA and RT ^2,3,5^. | - |
| Time | Total duration of MVPA in last week ^2^. | Total duration of MVPA in last week ^2^. |
| Sitting | Total duration of sitting in last week. Reverse scored to indicate that lower sitting time is beneficially associated with health ^6,7^. | Total duration of sitting in last week. Reverse scored to indicate that lower sitting time is beneficially associated with health ^6,7^. |
| **Sleep Health** |  |  |
| Daytime alertness | During the past month, how often have you had trouble staying awake while driving, eating meals, or engaging in social activity? Response options and scoring:  3 = Not during the past month; 2 = Less than once per week; 1 = Once or Twice per week,  0 = Three or more times per week ^8^. | - |
| Quality | During the past month, how would you rate your sleep quality overall? Response options and scoring:  3 = Very Good; 2 = Fairly Good; 1 = Fairly Bad;  0 = Very Bad ^8^. | During the past month, how would you rate your sleep quality overall? Response options and scoring:  3 = Very Good; 2 = Fairly Good; 1 = Fairly Bad;  0 = Very Bad ^8^. |
| Timing | When have you usually gone to bed? Response options: HH:MM  When have you usually gotten up in the morning? Response options: HH:MM  Midpoint of sleep calculated and classified as  0 = Midpoint not between 02:00-04:00; 10 = Midpoint between 02:00-04:00 ^8^. | - |
| Regularity | Average of two items:   1. I get out of bed at different times from day to day. 2. I go to bed at different times from day to day   Response options and scoring:  4 = never; 3 = rarely; 2 = sometimes; 1 = frequently; 0 = always ^9, 10^. |  |
| Efficiency | Sleep duration: How many hours of actual sleep do you get at night? (This may be different than the number of hours you spend in bed).  Time in bed: When have you usually gone to bed? When have you usually gotten up in the morning? Response options: HH:MM  Sleep efficiency: (sleep duration)/(time in bed) x 100 ^8^. | Sleep duration: How many hours of actual sleep do you get at night? (This may be different than the number of hours you spend in bed).  Time in bed: When have you usually gone to bed? When have you usually gotten up in the morning? Response options: HH:MM  Sleep efficiency: (sleep duration)/(time in bed) x 100 ^8^. |
| Duration | How many hours of actual sleep do you get at night? (This may be different than the number of hours you spend in bed). Classified based as: 0 = not recommended (<7 or >9 hours/night),  10 = recommended (7-9 hours/night).^8,11^ | How many hours of actual sleep do you get at night? (This may be different than the number of hours you spend in bed). Classified based as: 0 = not recommended (<7 or >9 hours/night),  10 = recommended (7-9 hours/night).^8,11^ |
| **Total Score** | Sum total of the 12 sub-dimensions scores. | Sum total of the 6 sub-dimensions scores. |

Notes.

1. Unless otherwise specified, all ASI-12 and ASI-6 sub-dimensions rescaled from 0-10 as follows: ((score – minimum score) / (score range))*10 with a higher score indicating a lower risk behaviour.
2. Moderate to Vigorous Intensity Physical Activity (MVPA) measured using the Active Australia Survey (3, 4).
3. Resistance training frequency measured using a single item measure of days per week (1, 2).
4. Based on evidence that accumulating a higher proportion of activity in vigorous intensity activity confers additional health benefits (5, 6). Studies use a classification of 30% or more of total activity accumulated in vigorous intensity activity and demonstrate that this confers additional health benefits.
5. Classification based on evidence that any participation in physical activity is better than none, and that participation in both MVPA and RT confers additional health benefits relative to participation in either (7)
6. Sitting time measured using the Workforce Sitting Time Questionnaire (8).
7. Lower volumes of sitting time are associated with reduced risk of all-cause and cardiovascular mortality (9-11).
8. Component measured using the Pittsburgh Sleep Quality Index (12).
9. Component measured using two items from the Sleep Hygiene Index (13).
10. Variability in sleep timing scored as the average of the two items.
11. Sleep duration classified as recommended or not recommended based on age appropriate sleep duration guidelines for adults aged 18-64 (14). Not recommended sleep duration scored as zero based on evidence the both shorter and longer than recommended sleep duration is adversely associated with health risks (14-16).

**References**

1. Murawski B, Plotnikoff RC, Rayward AT, Oldmeadow C, Vandelanotte C, Brown WJ, et al. Efficacy of an m-Health Physical Activity and Sleep Health Intervention for Adults: A Randomized Waitlist-Controlled Trial. Am J Prev Med. 2019;57(4):503-14.

2. Rayward AT, Murawski B, Duncan MJ, Holliday EG, Vandelanotte C, Brown WJ, et al. Efficacy of an m-Health Physical Activity and Sleep Intervention to Improve Sleep Quality in Middle-Aged Adults: The Refresh Study Randomized Controlled Trial. Annals of Behavioral Medicine. 2020.

3. Brown WJ, Bauman A, Chey T, Trost S, Mummery K. Comparison of surveys used to measure physical activity. Aust N Z J Public Health. 2004;28(2):128-34.

4. Brown WJ, Bauman A, Trost S, Mummery WK, Owen N. Test-retest reliability of four physical activity measures used in population surveys. Journal of Science and Medicine in Sport. 2004;7(2):205-15.

5. Gebel K, Ding D, Chey T, Stamatakis E, Brown WJ, Bauman AE. EFfect of moderate to vigorous physical activity on all-cause mortality in middle-aged and older australians. JAMA Internal Medicine. 2015.

6. Hamer M, Stamatakis E. Relative proportion of vigorous physical activity, total volume of moderate to vigorous activity, and body mass index in youth: the Millennium Cohort Study. Int J Obes. 2018;42(6):1239-42.

7. Stamatakis E, Lee IM, Bennie J, Freeston J, Hamer M, O'Donovan G, et al. Does Strength-Promoting Exercise Confer Unique Health Benefits? A Pooled Analysis of Data on 11 Population Cohorts With All-Cause, Cancer, and Cardiovascular Mortality Endpoints. American journal of epidemiology. 2018;187(5):1102-12.

8. Chau JY, van der Ploeg HP, Dunn S, Kurko J, Bauman AE. A tool for measuring workers' sitting time by domain: the Workforce Sitting Questionnaire. British journal of sports medicine. 2011;45(15):1216-22.

9. Ekelund U, Steene-Johannessen J, Brown WJ, Fagerland MW, Owen N, Powell K, et al. Does physical activity attenuate, or even eliminate the detrimental association of sitting time with mortality? A harmonised meta-analysis of data from more than one million men and women. The Lancet. 2016.

10. Ekelund U, Brown WJ, Steene-Johannessen J, Fagerland MW, Owen N, Powell KE, et al. Do the associations of sedentary behaviour with cardiovascular disease mortality and cancer mortality differ by physical activity level? A systematic review and harmonised meta-analysis of data from 850 060 participants. British journal of sports medicine. 2019;53(14):886-94.

11. Ekelund U, Tarp J, Steene-Johannessen J, Hansen BH, Jefferis B, Fagerland MW, et al. Dose-response associations between accelerometry measured physical activity and sedentary time and all cause mortality: systematic review and harmonised meta-analysis. Bmj. 2019;366:l4570.

12. Buysse DJ, Reynolds CF, 3rd, Monk TH, Berman SR, Kupfer DJ. The Pittsburgh Sleep Quality Index: a new instrument for psychiatric practice and research. Psychiatry Res. 1989;28(2):193-213.

13. Monk TH, Buysse DJ, Kennedy KS, Pods JM, DeGrazia JM, Miewald JM. Measuring sleep habits without using a diary: the sleep timing questionnaire. Sleep. 2003;26(2):208-12.

14. Hirshkowitz M, Whiton K, Albert SM, Alessi C, Bruni O, DonCarlos L, et al. National Sleep Foundation's updated sleep duration recommendations: final report. Sleep Health. 2015;1(4):233-43.

15. Itani O, Jike M, Watanabe N, Kaneita Y. Short sleep duration and health outcomes: a systematic review, meta-analysis, and meta-regression. Sleep Medicine. 2017;32(Supplement C):246-56.

16. Jike M, Itani O, Watanabe N, Buysse DJ, Kaneita Y. Long sleep duration and health outcomes: A systematic review, meta-analysis and meta-regression. Sleep Med Rev. 2017.

**Supplementary Table 3 Comparison of completers and non-completers in control and intervention groups pooled from SYNERGY and REFRESH trials**

|  | **Total** | **Non-completer** | **Completer** | **p-value** |
| --- | --- | --- | --- | --- |
|  | **N=325** | **N=50** | **N=275** |  |
| Age | 46.8 (10.0) | 43.4 (10.8) | 47.4 (9.7) | 0.008 |
| Sex |  |  |  | 0.30 |
| male | 61 (18.8%) | 12 (24.0%) | 49 (17.8%) |  |
| female | 264 (81.2%) | 38 (76.0%) | 226 (82.2%) |  |
| Education | 16.2 (2.8) | 15.9 (2.9) | 16.2 (2.8) | 0.40 |
| Occupation |  |  |  | 0.25 |
| Blue Collar | 9 (2.8%) | 2 (4.0%) | 7 (2.5%) |  |
| White Collar | 55 (16.9%) | 13 (26.0%) | 42 (15.3%) |  |
| Professional | 192 (59.1%) | 25 (50.0%) | 167 (60.7%) |  |
| Retired, Student, Home Duties, Other | 69 (21.2%) | 10 (20.0%) | 59 (21.5%) |  |
| Income/Yr |  |  |  | 0.66 |
| ≤$30,000 | 62 (19.1%) | 10 (20.0%) | 52 (18.9%) |  |
| $30,001-$50,000 | 40 (12.3%) | 3 (6.0%) | 37 (13.5%) |  |
| $50,001-$70,000 | 65 (20.0%) | 13 (26.0%) | 52 (18.9%) |  |
| $70,001-$100,000 | 72 (22.2%) | 12 (24.0%) | 60 (21.8%) |  |
| ≥$100,001 | 60 (18.5%) | 8 (16.0%) | 52 (18.9%) |  |
| don't know/prefer not to answer | 26 (8.0%) | 4 (8.0%) | 22 (8.0%) |  |
| Depressive Symptoms | 10.7 (8.1) | 13.5 (7.9) | 10.2 (8.0) | 0.006 |
| Anxiety Symptoms | 6.0 (5.5) | 7.2 (6.5) | 5.8 (5.2) | 0.078 |
| Stress Symptoms | 14.2 (6.6) | 15.9 (6.0) | 13.9 (6.7) | 0.044 |
| ASI-12 Score | 47.4 (10.9) | 45.1 (9.6) | 47.8 (11.1) | 0.11 |

Data are presented as mean (SD) for continuous measures, and n (%) for categorical measures.

Supplementary Table 4**. Spearman correlations between overall ASI-12 Score and ASI-12 sub-components at baseline**

| Variables | (1) | (2) | (3) | (4) | (5) | (6) | (7) | (8) | (9) | (10) | (11) | (12) | (13) |
| --- | --- | --- | --- | --- | --- | --- | --- | --- | --- | --- | --- | --- | --- |
| (1) ASI-12 Score | 1.00 |  |  |  |  |  |  |  |  |  |  |  |  |
| (2) MVPA Frequency Score | 0.36 | 1.00 |  |  |  |  |  |  |  |  |  |  |  |
| (3) RT Frequency Score | 0.38 | 0.15 | 1.00 |  |  |  |  |  |  |  |  |  |  |
| (4) MVPA Intensity Score | 0.44 | 0.27 | 0.36 | 1.00 |  |  |  |  |  |  |  |  |  |
| (5) Activity Type Score | 0.46 | 0.32 | 0.86 | 0.39 | 1.00 |  |  |  |  |  |  |  |  |
| (6) MVPA Duration Score | 0.38 | 0.63 | 0.22 | 0.46 | 0.39 | 1.00 |  |  |  |  |  |  |  |
| (7) Sitting Duration Score | 0.28 | -0.03 | 0.11 | 0.05 | 0.13 | 0.05 | 1.00 |  |  |  |  |  |  |
| (8) Sleep Alertness Score | 0.20 | 0.05 | -0.16 | -0.06 | -0.14 | -0.06 | -0.05 | 1.00 |  |  |  |  |  |
| (9) Sleep Quality Score | 0.33 | 0.09 | 0.03 | -0.00 | 0.07 | 0.06 | 0.09 | 0.08 | 1.00 |  |  |  |  |
| (10) Sleep Midpoint Score | 0.44 | 0.11 | 0.10 | 0.04 | 0.13 | 0.05 | -0.07 | -0.07 | -0.00 | 1.00 |  |  |  |
| (11) Sleep Timing Score | 0.11 | -0.01 | -0.01 | -0.02 | 0.00 | -0.01 | 0.06 | 0.08 | 0.00 | -0.20 | 1.00 |  |  |
| (12) Sleep Efficiency Score | 0.44 | 0.09 | -0.06 | 0.04 | 0.01 | 0.06 | 0.08 | 0.09 | 0.25 | 0.03 | -0.08 | 1.00 |  |
| (13) Sleep Duration Score | 0.60 | 0.06 | 0.06 | 0.09 | 0.06 | 0.09 | 0.10 | 0.04 | 0.11 | 0.09 | 0.02 | 0.46 | 1.00 |
|  | | | | | | | | | | | | | |

Supplementary Table 5. Results of mediation model examining effect of the intervention (baseline to 3 months) on ASI-6 and mental health outcomes at 3 months

| **Outcome** | **A(SE)** | **B(SE)** | **Direct(SE)** | **C(SE)** | **AB(SE)[95%CI]** |
| --- | --- | --- | --- | --- | --- |
| Depressive Symptoms | 2.38 (0.51)* | -0.16 (0.04)* | -0.18 (0.35) | -0.57 (0.35) | -0.39 (0.14) [-0.71,-0.16] |
| Anxiety Symptoms | 2.39 (0.51)* | -0.07 (0.03)* | -0.18 (0.22) | -0.35 (0.22) | -0.17 (0.08) [-0.35,-0.04] |
| Stress Symptoms | 2.37 (0.51)* | -0.19 (0.04)* | -0.20 (0.31) | -0.64 (0.31)* | -0.44 (0.14) [-0.76,-0.22] |
| QOL Physical Score | 2.39 (0.51)* | 0.01 (0.05) | 0.31 (0.45) | 0.35 (0.43) | 0.03 (0.13) [-0.21,0.31] |
| QOL Mental Score | 2.40 (0.51)* | 0.31 (0.07)* | 0.34 (0.57) | 1.10 (0.57) | 0.75 (0.24) [0.36,1.31] |
| Energy & Fatigue | 2.39 (0.51)* | 0.45 (0.11)* | 0.90 (0.91) | 1.98 (0.90)* | 1.08 (0.36) [0.49,1.92] |

Notes. In all models a path adjusted for study (Synergy, REFRESH) and baseline value of mediator, b path adjusted for study (Synergy, REFRESH) and baseline value of mediator and outcome. * indicates p<0.05

Supplementary Table 6. Results of mediation model examining effect of the intervention (baseline to 3 months) on ASI-6 and mental health outcomes at 6 months

| **Outcome** | **A(SE)** | **B(SE)** | **Direct(SE)** | **C(SE)** | **AB(SE)[95%CI]** |
| --- | --- | --- | --- | --- | --- |
| Depressive Symptoms | 1.79 (0.58)* | -0.19 (0.05)* | -0.42 (0.43) | -0.76 (0.44) | -0.34 (0.15) [-0.74,-0.11] |
| Anxiety Symptoms | 1.82 (0.58)* | -0.13 (0.04)* | -0.24 (0.29) | -0.48 (0.29) | -0.24 (0.13) [-0.65,-0.08] |
| Stress Symptoms | 1.80 (0.58)* | -0.28 (0.05)* | -0.47 (0.40) | -0.97 (0.42)* | -0.50 (0.20) [-0.98,-0.19] |
| QOL Physical Score | 1.74 (0.58)* | 0.14 (0.06)* | 0.78 (0.50) | 1.03 (0.50)* | 0.25 (0.15) [0.04,0.66] |
| QOL Mental Score | 1.79 (0.58)* | 0.24 (0.08)* | 0.10 (0.66) | 0.53 (0.66) | 0.43 (0.22) [0.12,1.01] |
| Energy & Fatigue | 1.77 (0.58)* | 0.50 (0.14)* | 2.53 (1.16) | 3.41 (1.17)* | 0.88 (0.44) [0.25,2.05] |

Notes. In all models a path adjusted for study (Synergy, REFRESH) and baseline value of mediator, b path adjusted for study (Synergy, REFRESH) and baseline value of mediator and outcome. * indicates p<0.05

| Supplementary Table 7. Comparison of ASI scores between those who improved or reported stable or worsening symptoms | | | | | | |
| --- | --- | --- | --- | --- | --- | --- |
|  | **Stable/Decrease** | | **Improved** | |  |  |
|  | **N** | **M(SD)** | **N** | **M(SD)** | **Difference (95%CI)** | **d (95%CI)** |
|  | **Depression** |  |  |  |  |  |
| **ASI Change 0-3mo** | 210 | 6.81 (13.75) | 61 | 10.92 (14.86) | -4.11 (-8.12, -0.10) | -0.29 (-0.58, -0.01) |
|  | **Anxiety** |  |  |  |  |  |
| **ASI Change 0-3mo** | 220 | 8.14 (13.33) | 52 | 5.97 (16.85) | 2.17 (-2.10, 6.43) | 0.15 (-0.15, 0.46) |
|  | **Stress** |  |  |  |  |  |
| **ASI Change 0-3mo** | 210 | 6.92 (13.41) | 52 | 10.45 (15.89) | -3.52 (-7.51, 0.46) | -0.25 (-0.53, 0.03) |

Supplementary table 8. Risk of Bias Assessment for included studies based on Cochrane ROB

|  | **Randomization Process** | **Deviations from intended interventions** | **Missing outcome data** | **Measurement of the outcome** | **Selection of Reported Outcome** | **Overall Bias** |
| --- | --- | --- | --- | --- | --- | --- |
| Refresh Study^1^ | Low | Low | Low | Some concerns | Low | Low |
| Synergy Study^2^ | Low | Low | Low | Some concerns | Low | Low |

Notes.

1. Rayward, A. T., Murawski, B., Duncan, M. J., Holliday, E. G., Vandelanotte, C., Brown, W. J., & Plotnikoff, R. C. (2020). Efficacy of an m-Health Physical Activity and Sleep Intervention to Improve Sleep Quality in Middle-Aged Adults: The Refresh Study Randomized Controlled Trial. Annals of Behavioral Medicine. doi:10.1093/abm/kaz064
2. Murawski, B., Plotnikoff, R. C., Rayward, A. T., Oldmeadow, C., Vandelanotte, C., Brown, W. J., & Duncan, M. J. (2019). Efficacy of an m-Health Physical Activity and Sleep Health Intervention for Adults: A Randomized Waitlist-Controlled Trial. American Journal of Preventive Medicine, 57(4), 503-514. doi:10.1016/j.amepre.2019.05.009
